# Analysis of intestinal bacterial community diversity of adult *Dastarcus helophoroides*

**DOI:** 10.1093/jis/14.1.114

**Published:** 2014-08-12

**Authors:** Z. Q. Zhang, C. He, M. L. Li

**Affiliations:** 1 Laboratory of Forestry Pests Biological Control, College of Forestry, Northwest Agriculture and Forestry University, Yangling, Shaanxi, 712100, China; 2 Wuwei academy of Forestry Sciences, WuWei, Gansu, 733000, China

**Keywords:** PCR-DGGE assay, culture-dependent technique

## Abstract

Polymerase chain reaction denaturing gradient gel electrophoresis (PCR-DGGE), and a culture-dependent technique were used to study the diversity of the intestinal bacterial community in adult *Dastarcus helophoroides* (Fairmaire) (Coleoptera: Bothrideridae). Universal bacterial primers targeting 200 bp regions of the *16S rDNA* gene were used in the PCR-DGGE assay, and 14 bright bands were obtained. The intestinal bacteria detected by PCR-DGGE were classified to *Enterococcus* (Lactobacillales: Enterococcaceae), *Bacillus* (Bacillales: Bacillaceae), *Cellvibrio* (Pseudomonadales: Pseudomonadaceae), *Caulobacter* (Caulobacterales: Caulobacteraceae), and uncultured bacteria, whereas those isolated by the culture-dependent technique belonged to *Staphylococcus* (Bacillales: Staphylococcaceae), *Pectobacterium* Enterobacteriales: Enterobacteriaceae), and *Enterobacter* (Enterobacteriales: Enterobacteriaceae). These intestinal bacteria represented the groups Lactobacillales ( *Enterococcus* ), Pseudomonadales ( *Cellvibrio* ), Caulobacterales ( *Caulobacter* ), Bacilli ( *Bacillus* and *Staphylococcus* ), and Gammaproteobacteria ( *Pectobacterium* and *Enterobacter* ). Our results demonstrated that PCR-DGGE analysis and the culture-dependent technique were useful in determining the intestinal bacteria of *D. helophoroides* and the two methods should be integrated to characterize the microbial community and diversity.

## Introduction

Many insects are inhabited by diverse communities of microorganisms. It is possible that the number of microbes in most insects is larger than the number of somatic cells ( Campbell 1990 ). The intestinal microbial bacteria in insects have been shown to play important roles, such as providing vitamins, aiding in fat and carbohydrate metabolism, preventing the invasion of external bacteria, and promoting the function of the immune system ( Eutick et al. 1978 , Abe et al. 2000 , Suchodolski and Ruaux 2004 ). Insects’ guts offer many niches for bacteria, while the insects take advantage of the bacterial metabolism and the adaptability of prokaryotes ( Dillon and Dillon 2004 ). For example, extensive research has revealed the symbiotic relationship between termites and bacteria. Microbes provide carbon, nitrogen, and other nutrient sources to their host termites, and termites can no longer live without them ( Hongoh et al. 2003 ). The analysis of intestinal bacteria was performed by culture-dependent and molecular methods. The culture-dependent technique defined the gut microbes by phenotypic characterization (morphology, immunology, and physiological-biochemical reaction), but unculturable bacteria were largely ignored ( Lysenko 1985 ). Molecular biology methods allowed extracting the total genomic DNA of bacteria directly from samples and then sequencing and analyzing the DNA to characterize the bacteria species composition and abundance ( Yu et al. 2008 ).

Coupled polymerase chain reaction and denaturing gradient gel electrophoresis (PCR-DGGE) and other modern molecular biological technologies have been applied to study the microbial diversity and dominant species in many insects, such as *Reticulitermes speratus* Kollbe (Isoptera: Rhinotermitidae) ( Hongoh et al. 2003 ), *Hepialus gonggaensis* Fu & Huang (Lepidoptera: Hepialidae) ( Zhuo 2005 ), *Bombyx mori* L. (Lepidoptera: Bombycidae) ( Xiang et al. 2007 ), *Anopheles sinensis* Wiedemann (Diptera: Culicidae) ( Wang 2008 ), and *Costelytra zealandica* (White) (Coleoptera: Melolonthidae) ( Zhang and Jackson 2008 ), but few reports are available on the intestinal bacteria of *Dastarcus helophoroides* (= *longulus* ) (Fairmaire) (Coleoptera: Bothrideridae). This parasitic beetle is an important natural enemy of longhorned beetles ( Ogura et al. 1999 ) and is therefore an important experimental insect.

Amann et al. (1995) indicated that only 0.1– 15% of microbes from natural environments could grow in artificial media. Hence, results from culture-dependent methods only partially reflected the real microbial communities and restricted our past knowledge of microbial ecology. For example, when culture-dependent methods were applied to assay the gut microbial community of *H. gonggaensis* larvae, 12 bacteria were isolated ( Zhuo et al. 2004 ). In contrast, by using a PCR-DGGE assay, Zhuo (2005) separated 76 bands that all represented different bacteria from this insect. Muyzer et al. (1993) were among the first researchers who used DGGE to analyze the genetic diversity of microorganisms in algal-fungal and bacterial biofilms, and their results indicated that this technique offered advantages over culture-dependent methods in revealing natural microbial communities.

The aim of this study was to analyze the diversity of the intestinal bacterial community in adult *D. helophoroides* by using culture-dependent and PCR-DGGE techniques and to compare the results obtained by both methods.

## Materials and Methods

### Sample collection

Adults of *D. helophoroides* were obtained from the Laboratory of Forest Pest Biological Control, College of Forestry, Northwest Agriculture and Forestry University, China. All insects were maintained and reared in controlled incubators at 25 ± 1°C, 50-70% relative humidity (RH), and a photoperiod of 10:14 (L:D) on an artificial diet ( Zhang et al. 2014 ). The adult beetles were starved for 12 hr to allow elimination of the food bolus. The digestive tracts were carefully removed from the abdomen by using sterile dissecting needles, and five guts were pooled and crushed gently with a pestle in liquid nitrogen.

### Genomic DNA extraction and *16S rDNA* amplification

A modified cetyltrimethyl ammonium bromide method was used for genomic DNA extraction ( Calderón-Cortés et al. 2010 ). The extracted genomic DNA was electrophoresed through a 1.0% TAE (40 mM Tris, 20 mM acetic acid, 1.0 mM Na _2_ -EDTA, 1.0% agarose) gel for detection of the extracted genomic DNA.

The universal primers 27mf (5′-AGA GTT TGA TCM TGG CTC AG-3′) and 1492r (5′-TAG GGY TAC CTT GTT ACG ACT T-3′) were used to amplify *16S rDNA* genes of the intestinal bacteria ( Weisburg et al. 1991 ). The primers 338GC (5ʹ-CGC CCG CCG CGC CCC GCG CCC GGC CCG CCG CCG CCG CCG CAC TCC TAC GGG AGG CAG CAG-3ʹ) and RP534 (5ʹ-ATT ACC GCG GCT GCT GG-3ʹ) were used to amplify the *16S rDNA* V3 regions with PCR-DGGE, and 341F (5ʹ-CCT ACG CGA GGC AGC AG-3ʹ) and RP534 were used to sequence the isolated products ( Muyzer et al. 1993 ). The PCR amplification was performed in a final volume of 50 µL, consisting of 25 µL 2× Es Taq Master Mix (withdye; CWBIO, www.cwbiotech.com ), 19 µL RNase-free H _2_ O, 2 µL of each primer, and 2 µL template DNA. The PCR was performed in a DNA Engine Dyas Peltier Thermal Cycler (Bio-Rad Life Science Product, Hercules, CA). The amplification program (designed by lab members) was as follows: an initial denaturation at 95°C for 5 min; 29 cycles of denaturation at 94°C for 1 min, annealing at 55°C for 30 sec, extension at 72°C for 90 sec, and a final extension at 72°C for 10 min. The amplification products were purified with a universal DNA purification kit (TIANGEN Biotech, Beijing, China).

### DGGE analysis

The PCR products (30 µL) were separated and analyzed by DGGE on an 8% polyacrylamide gel with a denaturant-gradient of 35-60% (100% defined as 7 M solid urea and 40% deionized formamide [v/v]) in a Dcodek ^TM^ Universal Mutation Detection System (Bio-Rad). The electrophoresis was carried out in 1× TAE buffer (40 mM Tris, 20 mM acetic acid, 1.0 mM Na _2_ -EDTA) at 35 V and 60°C for 17 hr ( Fischer and Lerman 1983 ). The DGGE gel was stained in ethidium bromide for 10–20 min and visualized by UV light on a Bio-Rad 2000 GEL imaging system. Bright bands were excised from the gel with a sterile scalpel blade and eluted in 30 µL sterile distilled H _2_ O at 4°C for 12 hr. The mixture was centrifuged at 10,000 rpm (10,000 × *g* ) for 5 min at 4°C ( Kowalchuk et al. 1998 ). The supernatant was used as DNA template for *16S rDNA* V3 amplification with the primers 341F and RP534 and the program described in the section “Genomic DNA extraction and *16S rDNA* amplification.” The PCR products were electrophoresed through a 1.5% TAE agarose gel and purified with the universal DNA purification kit (TIANGEN) for sequencing (Sangon Biotech, Shanghai, China).

### Intestinal bacteria isolation and cultivation

The guts of five adult beetles were pooled and ground with a sterilized pestle. The sample was homogenized with 1 mL sterilized distilled H _2_ O and then diluted to serial 10-fold dilutions with sterilized distilled water (i.e., dilution 1 = undiluted stock, dilution 2 = 1/10, dilution 3 = 1/100, and dilution 4 = 1/1,000). Aliquots (100 µL) of the four dilutions were spread uniformly on solid NA medium (3 g beef extract, 10 g peptone, 10 g glucose, 16 g agar, water added to a final volume of 1,000 mL). Three replicate plates were incubated separately at 37°C and three at 27°C for 48 hr. The different bacteria colonies were selected, transferred to new NA plates, and incubated for 48 hr at 37°C and 27°C, respectively. The pure cultured strains served as DNA templates for PCR amplification with the primers 27mf and 1492r. The PCR products were sent to Sangon (Shanghai, China) for sequencing.

### Phylogenetic analysis

Sequences were compared with known sequences listed in the GenBank nucleotide sequence database. The BLAST search option of the National Center for Biotechnology Information (NCBI) ( www.ncbi.nlm.nih.gov/ ) was used to search for close evolutionary relatives in the GenBank database. Neighbor-joining trees of the sequences were constructed by using MEGA 5.0 ( Graham et al. 2007 ).

## Results

### The DGGE pattern and phylogenetic analysis of intestinal bacteria in *D. helophoroides*

**Figure 1.**
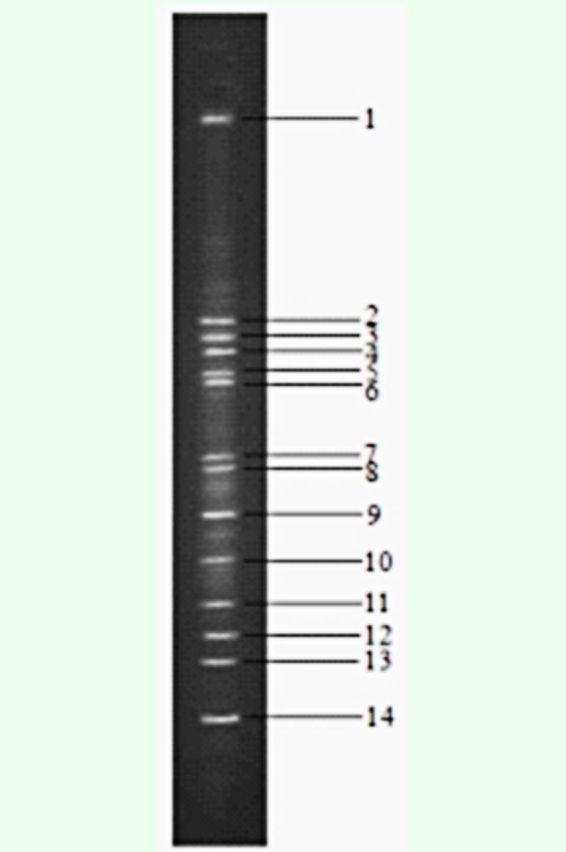
Detail of the ethidium bromide–stained 16S *rDNA* DGGE profiles. The following genera were identified from the gut of *Dastarcus helophoroides: Enterococcus* (bands 2, 3, 4), *Cellvibrio* (bands 5, 6), *Bacillus* (bands 7, 8, 14), *Caulobacter* (band 11), and uncultured bacteria (bands 1, 9, 10, 12, 13). High quality figures are available online.

**Figure 2.**
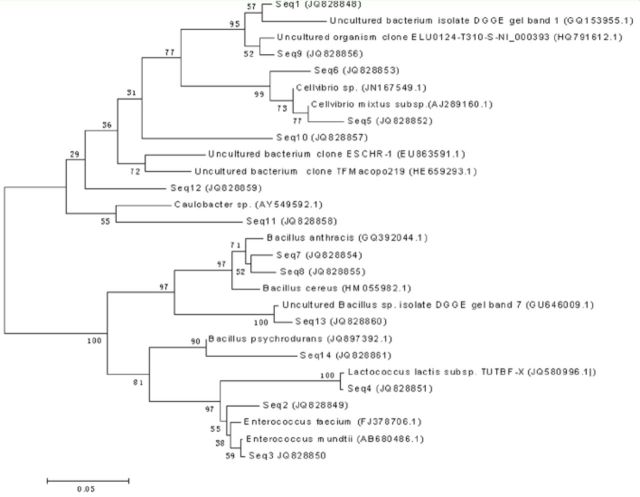
The phylogenetic tree of *Dastarcus helophoroides* intestinal bacteria detected in *D. helophoroides* by the PCR-DGGE method. Sequences with high species homology in GenBank were Seq2 *(Enterococcus faecium),* Seq3 *(Enterococcus mundtii),* Seq4 *(Lactococcus lactis),* Seq5 *(Cellvibrio mixtus),* Seq6 *(Cellvibrio* sp *.* ), Seq7 *(Bacillus anthracis),* Seq8 *(Bacillus cereus),* Seq14 *(Bacillus psychrodurans),* Seq 1 1 *(Caulobacter* sp *.* ), and uncultured bacteria (Seq 1, Seq9, Seq 10, Seq 1 2, Seq 1 3). Numbers at nodes indicate bootstrap values out of 100 bootstrap resamplings. High quality figures are available online.

Bacterial genomic DNA served as templates for PCR amplification with universal bacterial primers 338GC and RP534, and the target segments of 200 bp were obtained. For phylogenetic identification, sequences were compared with *16S rDNA* sequence information of known bacteria listed in the GenBank database. The DGGE bands of *D. helophoroides* intestinal bacteria are shown in Fig. 1 , and the results of the phylogenetic analysis are shown in Fig. 2 , revealing the presence of a variety of different genera. Through neighbor-joining analysis, 14 bacterial sequences were divided into two main groups, namely Firmicutes and Proteobacteria. The BLAST analysis of these sequences ( Table 1 ) revealed that Seq2, Seq3, and Seq4 shared high similarity values to *Enterococcus* (Lactobacillales: Enterococcaceae); Seq5 and Seq6 were similar to *Cellvibrio* (Pseudomonadales: Pseudo-monadaceae); Seq7, Seq8, and Seq14 were most closely related to *Bacillus* (Bacillales: Bacillaceae); and Seq11 was similar to *Caulobacter* (Caulobacterales: Caulobacteraceae). Seq1, Seq9, Seq10,Seq12, and Seq13 had high levels ofidentity (91–99%) with uncultured bacte riumsequences (GQ153955.1, HQ791612.1, HE659293.1, EU863591.1, and GU646009.1, respectively) in NCBI’s GenBank. All of the 14 sequences were submitted to GenBank with the accession numbers JQ828848-JQ828861.

**Table 1.**
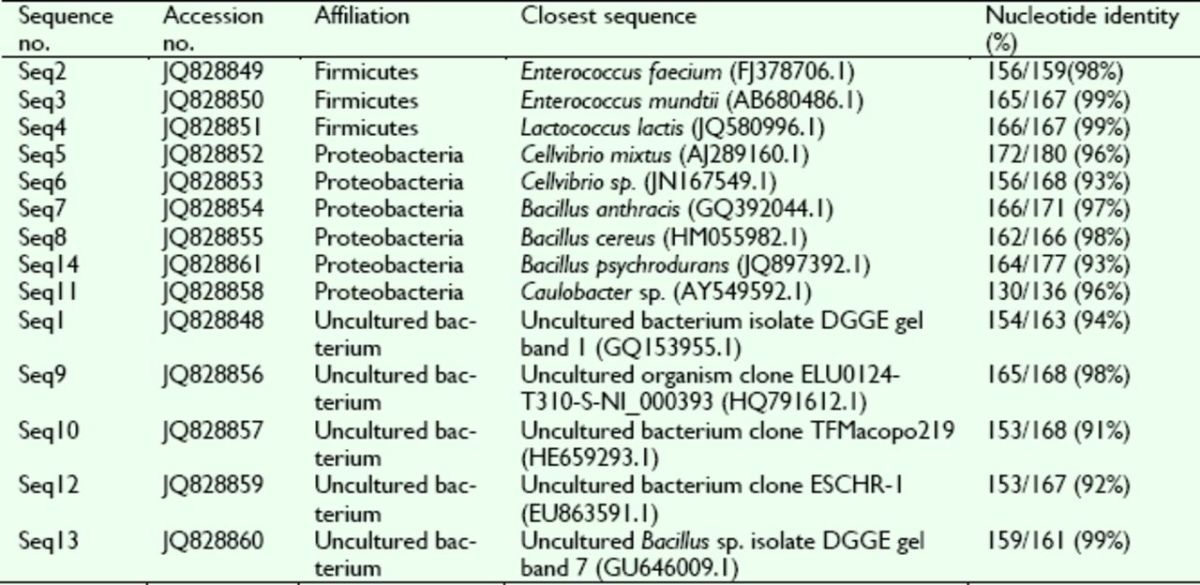
*16S rDNA* sequences of *Dastarcus helophoroides* intestinal bacteria identified by the PCR-DGGE method.

### Culture-dependent identification of intest inal bacteria in *D. helophoroides*

**Figure 3.**
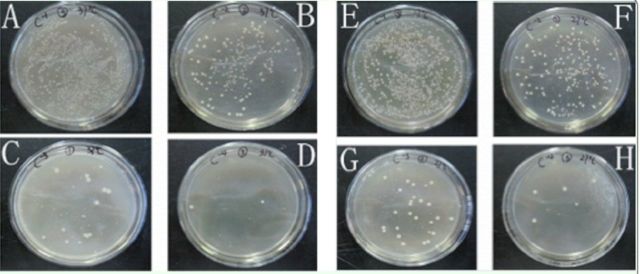
Selected culture plates of different dilutions of *Dastarcus helophoroides* gut homogenates maintained at two temperatures. A, stock at 37°C; B, 10-fold dilution at 37°C; C, 100-fold dilution at 37°C; D, 1,000-fold dilution at 37°C; E, stock at 27°C; F, 10-fold dilution at 27°C; G, 100-fold dilution at 27°C; H, 1,000-fold dilution at 27°C. High quality figures are available online.

**Figure 4.**
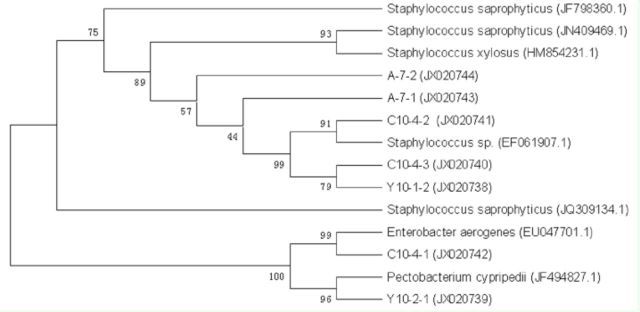
Phylogenetic tree of intestinal bacteria of *Dastarcus helophoroides* obtained by the culture-dependent method. The following species were identified: *Staphylococcus* sp. (Y10-1-2, A-7-1, A-7-2, C10-4-2, C10-4-3), *Pectobacterium cypripedi* (Y10-2-1), and *Enterobacter aerogenes* (C10-4-1). Numbers at nodes indicate bootstrap values out of 100 bootstrap resamplings. High quality figures are available online.

In the culture-dependent experiment, each iso lated bacterial strain was separated by different color and size. The 10-fold dilutions of gut homogenates produced bacterial colo nies at densities that were most suitable for

isolation ( Fig. 3 ). Ten bacterial strains were isolated from the NA culture medium, and seven *16S rDNA* sequences were obtained and submitted to the GenBank nucleic acid sequence database under the accession numbers JX020738-JX020744. BLAST analysis of the seven sequences revealed that Seq1 (Y10-1-2) shared 97% identity (799/824 nt) with *Staphylococcus saprophyticus* Fairbrother (Bacillales: Staphylococcaceae), Seq2 (Y10-2-1) shared 98% identity (976/993 nt) with *Pantoea* sp. (Enterobacteriales: Enterobacteri-aceae), Seq3 (C10-4-3) shared 98% identity (966/981 nt) with *S. saprophyticus* , Seq4 (C10-4-2) shared 90% identity (743/825 nt) with *Staphylococcus* sp., Seq5 (C10-4-1) shared 99% identity (935/939 nt) with *Enter-obacter aerogenes* Hormaeche and Edwards (Enterobacteriales: Enterobacteriaceae), Seq6 (A-7-1) shared 99% identity (1025/1031 nt) with *Staphylococcus xylosus* Schleifer and Kloos (Bacillales: Staphylococcaceae), and Seq7 (A-7-2) shared 99% identity (1010/1014 nt) with *S. saprophyticus* . The neighbor- joining method of MEGA 5 was used to construct a phylogenetic tree ( Fig. 4 ). The results showed that three genera were isolated. *Staphylococcus* (Bacillales: Staphylococca-ceae) was the dominant genus (five out of seven isolates＝71%), whereas isolates C10-4-1 and Y10-2-1 belonged to the genera *Pectobacterium* (Enterobacteriales: Enterobacteriaceae) and *Enterobacter* (Enter-obacteriales: Enterobacteriaceae), respectively.

### Comparison of the intestinal bacteria obtained by two methods

Twelve known genera of bacteria were isolated by two methods in this study. Table 2 shows that 67% (eight genera) were isolated by PCR-DGGE, whereas 33% (four genera) were isolated by the culture-dependent technique. The common order of bacteria obtained by the two methods was Enterobacteriales. Caulobacterales, Lactobacillales, and Pseudomonadales were obtained only by the PCR-DGGE assay, while the order Bacillales was detected only by the culture-dependent technique. The genera *Bacillus* and *Enterococcus* showed high occurrence in the PCR-DGGE assay, and *Staphylococcus* was dominant in the culture-dependent technique.

**Table 2.**
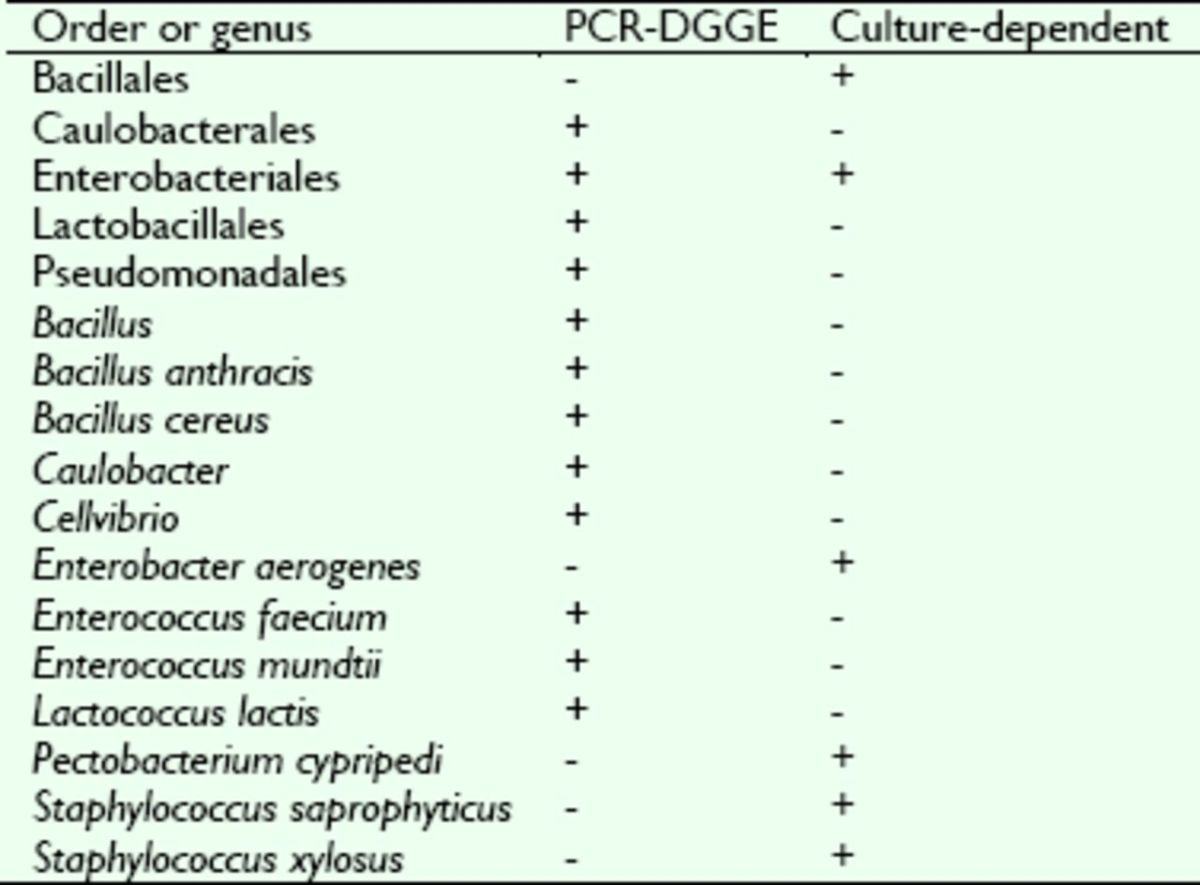
Comparison of *Dastarcus helophoroides* intestinal bacteria identified by different methods.

+, the detected order/genera; -, the undetected order/genera.

## Discussion

A culture-dependent method and a nucleic-acid based technique were used to reveal the intestinal bacterial community structure of *D. helophoroides.* Twelve genera of intestinal bacteria were isolated, and different genera were obtained by the two methods. Four genera of intestinal bacteria were isolated by the culture-dependent technique, among which *Staphylococcus* was the predominant genus, whereas eight genera were detected by the PCR-DGGE assay. Hence, the PCR-DGGE indicated a greater bacterial diversity than the culture-dependent technique. If we had applied the culture-dependent technique alone, we would have concluded that the intestinal bacterial diversity in *D. helophoroides* was relatively low.

Moreover, the two methods showed different predominant bacteria. Most bacteria detected by the molecular technique cannot be isolated and cultured by traditional culture methods, and several bacteria obtained by the culture-dependent technique were detected at a low abundance in the molecular biology technique. Some highly abundant bacteria could not be cultivated because the culture conditions were not suitable for their growth. In contrast, less abundant bacteria could grow rapidly and become the dominant bacteria in a short time period. The PCR-DGGE assay was expected to separate the bacteria by single-base differences, as denaturation temperature and gel concentration would influence the migration rates of the PCR products. Often, bands at different locations in the denaturing gel represent different bacteria. However, it has been reported that different bands could represent the same bacteria or that bands at the same location could be identified as different bacteria ( Jackson et al. 2000 , Suchodolski and Ruaux 2004 ). In our research, 17 bands were excised from the gel and 14 of them could be sequenced. Some of these sequences were classified to the same genera, with Seq2, Seq3, and Seq4 belonging to *Enter ococcus;* Seq5 and Seq6 belonging to *Cellvibrio;* and Seq7, Seq8, and Seq14 belonging to *Bacillus.*

Most of the bacteria that were isolated in this study have been found in many insects. Zhuo et al. (2004) examined the intestinal flora of the hepialid *H. gonggaensis* with a culture-dependent method and found that *Staphylo coccus* sp *.* was the predominant community member. Species of the genus *Staphylococcus* which are Gram-positive bacteria and mostly nonpathogenic, often can be found in the guts of insects. Liu et al. (2007) isolated 16 bacte ria strains from the gut of adults (male and female) of *Locusta migratoria manilensis* (Meyen) (Orthoptera: Acrididae) and classified them as *Serratia, Yokenella, Enterobacter, Citrobacter, Salmonella, Kluy-vera, Klebsiella* (Enterobacteriales: Enterobacteriaceae); *Brachybacterium* (Acti- nomycetales: Dermabacteraceae); *Microbacterium, Clavibacter* (Actinomy-cetales: Microbacteriaceae); *Paenibacillus* (Bacillales: Paenibacillaceae); *Actinobacillus* (Pasteurellales: Pasteurellaceae); *Acinetobacter* (Pseudomonadales: Moraxellaceae); and *Staphylococcus.*Yuan et al. (2006) discovered that the main bacteria in the intestinal tract of the silkworm *B. mori* were *Arthrobacter* (Ac-tinomycetales: Micrococcaceae), *Lactobacillus* (Lactobacillales: Lactobacil-laceae), *Escherichia* (Enterobacteriales:Enterobacteriaceae), *Pseudomonas* (Pseudo-monadales: Pseudomonadaceae), *Bacillus,* and *Staphylococcus.*Yang et al. (2006) analyzed the different bacteria in termites and found that they were *Streptococcus* (Lactobacillales: Streptococcaceae), *Bacteroides* (Bacteroi- dales: Bacteroidaceae), *Bacillus, Staphylococcus,* and *Enterobacter.*Wang (2008) obtained 28 different DGGE bands of bacteria from the guts of larvae of the mosquito *A. sinensis,* and Gammaproteobacteria, Flavobacteria, Actinobacteria, Betaproteobac-teria, and Firmicutes were observed. Zhang (2007) found *Aureobacterium* (Actinomy-cetales: Microbacteriaceae), *Bacillus sphaericus* Meyer and Neide (Bacillales: Bacillaceae), *Microbacterium* Orla-Jensen, *Bacillus megaterium* de Bary (Bacillales: Bacillaceae), and *Cnrtobacterium* Yamada in the intestines of *Tenebrio molitor* L. (Coleoptera: Tenebrionidae). Broderick et al. (2004) investigated the intestinal bacteria of larvae of *Lymantria dispar* L. (Lepidoptera: Lymantriidae), and their results showed that *Enterococcus* was the dominant bacterium.

Hence, we expected that the intestinal bacteri al community of *D. helophoroides* would have many similarities with those found in species of Orthoptera, Hymenoptera, Lepidoptera, and especially Coleoptera. These insects have different living conditions, habits, and food sources, but most of them harbor common bacteria in their guts. The ubiquitous presence indicates that these intestinal bacteria are not influenced by feeding habits or food sources of their hosts and that they are the intrinsical gut bacteria of insects. Most bacteria isolated in this research were the same as the intestinal bacteria reported before, such as *Bacillus, En terococcus, Staphylococcus,* and *Enterobacter.* However, *Cellvibrio, Caulobacter,* and *Pectobacterium* were particular.We found that *Cellvibrio* was a bacterium that could decompose cellulose, *Pectobacterium* was a plant-putrefying bacterium, and *Caulobacter* was found mainly in freshwater and soil. It is possible that these bacteria originated from the artificial diet used to rear the adults of *D. helophoroides.*

The comparison of the culture-dependent technique and the PCR-DGGE assay used in our study indicated that both methods had shortcomings in isolating the intestinal bacterial community of *D. helophoroides.* Limitations, such as preferential amplification of *16S rDNA* of some bacterial taxa and identical electrophoretic migration of sequences with multiple differences, could lead to an underestimation of bacterial diversity in DGGE community fingerprintings ( Farrelly et al. 1995 , Polz and Cavanaugh 1998 , Jackson et al. 2000 ). Whereas *Staphylococcus* was the predominant bacterium obtained with the culture-dependent technique, *Enterococcus* and *Bacillus* were obtained when the PCR-DGGE assay was used. The short sequences (max. 200 bp) obtained by PCR-DGGE may have affected the resolution of the taxa in the analysis ( Asakawa and Kimura 2008 ). Furthermore, although universal primers were used for amplification of a conserved region in the bacterial genome, the presence of a wide variety of DNA templates in the reaction could have affected the results. The PCR-DGGE method likely could not detect microorganisms present at a level lower than 1% of the total microbial population ( Felske et al. 1998 , Zoetendal et al. 1998 ).

In conclusion, the culture-dependent technique yielded different bacteria than the molecular method. Therefore, the two methods should be combined to obtain the complete information when the microbial community structure and diversity in the digestive tracts of insects are investigated.
